# Comparison of Cobas Plasma Separation Cards and Whatman filter paper cards versus conventional serum samples for anti-*Trypansoma cruzi* antibody detection

**DOI:** 10.1371/journal.pntd.0014554

**Published:** 2026-08-21

**Authors:** Aroa Silgado, Alejandro Mediavilla, Patricia Martínez-Vallejo, Carles Rubio-Maturana, Lidia Goterris, Pau Bosch-Nicolau, Inés Oliveira-Souto, Núria Serre-Delcor, Israel Molina, Elena Sulleiro

**Affiliations:** 1 Department of Microbiology, Vall d’Hebron University Hospital, Universitat Autònoma de Barcelona, PROSICS Barcelona, Spain; 2 Centro de Investigación Biomédica en Red de Enfermedades Infecciosas (CIBERINFEC), Instituto de Salud Carlos III, Madrid, Spain; 3 Department of Infectious Diseases, Vall d’Hebron University Hospital. Universitat Autònoma de Barcelona, PROSICS Barcelona, Spain; Centro de Pesquisa Gonçalo Moniz-FIOCRUZ/BA, BRAZIL

## Abstract

**Background:**

Diagnosis of chronic *Trypanosoma cruzi* infection relies on serological techniques using invasive blood samples (serum or plasma), which can be impractical in remote areas. Dried blood spot (DBS) sampling offer advantages over invasive samples in terms of ease of collection (finger prick), transport and storage. This study aimed to compare the performance of anti-*T. cruzi* antibody detection from DBS samples collected on two different devices: Whatman cards and Cobas Plasma Separation Cards (PSC).

**Methodology/principal findings:**

A prospective study using 382 paired serum and whole blood samples was conducted. Whole blood were used to obtain DBS collected on Whatman and PSC cards. All samples were tested for IgG anti-*T. cruzi* using the Elecsys Chagas assay, with serum serving as the gold standard. Both DBS collection systems showed high concordance with serum samples (>96%). However, antibody index values detected in DBS samples were lower than those detected in serum samples (p < 0.05). Qualitative results from DBS were highly concordant between the collection systems, with only two discrepancies (2/382), both positive on Whatman and negative on PSC. Antibody index values from Whatman-collected samples were higher than those from PSC-collected samples (median 96.45 vs. 63.33; p < 0.05).

**Conclusions/significance:**

DBS sampling is a feasible alternative to invasive blood collection, particularly in settings with limited access to health systems. Both DBS-collection devices showed high diagnostic accuracy; however, the lower antibody index values from PSC-collected samples may compromise the sensitivity of the technique in samples with a low antibody index.

## Author summary

Chagas disease, caused by *Trypanosoma cruzi*, is a long-lasting infection that affects millions of people, mainly in Latin America, and can cause serious heart and digestive complications if it is not diagnosed and treated in time. Diagnosis usually relies on blood drawn from a vein, which can be difficult to collect, store, and transport in remote or resource-limited settings. We explored a simpler alternative based on a few drops of blood obtained by finger prick and dried on filter paper cards. In our study, we compared two types of dried blood spot collection devices with reference serum testing to detect antibodies against *T. cruzi*. We found a high level of agreement between dried blood spot samples and conventional blood samples, supporting their reliability as a minimally invasive and logistically simpler approach. Overall, our findings show that dried blood spot sampling is a practical and accurate strategy that could help expand access to diagnosis in underserved communities.

## Introduction

Chagas disease (CD), caused by the protozoan *Trypanosoma cruzi*, remains a neglected tropical disease with an estimated 7 million people infected worldwide and causing more than 10.000 deaths annually. Although CD is endemic in 21 continental Latin American countries, an increasing number of cases are being reported outside the endemic area [1].

Despite many efforts to control de disease, CD suffers from a high rate of underdiagnosed cases, with limited or no access to diagnostic tools, particularly in rural areas from endemic regions [2]. The diagnostic challenges in remote areas, where the prevalence is higher, lead to limited access to adequate clinical management, thereby significantly increasing the risk of developing severe complications associated with the infection.

Current diagnostic protocols for the chronic phase of *T. cruzi* infection rely on the performance of at least two different serological assays for the detection of anti-*T. cruzi* antibodies [3]. Indirect immunofluorescence assay, indirect haemagglutination or enzyme-linked immunosorbent assay (ELISA) are commonly used [4]. However, a serological diagnosis using only one highly sensitive and specific test –chemiluminescent immunoassay– has been proved to be useful [5,6]. Although this diagnostic algorithm provides high sensitivity and specificity, its implementation in resource-poor settings is hindered by the need for skilled personnel and an invasive whole blood sample collected by venipuncture is required [7]. Venous blood samples require centrifugation and reliable cold-chain storage [8], conditions rarely met in remote settings. Dried blood spot (DBS) from finger stick sampling provides a practical, less invasive alternative for blood collection in serological testing, overcoming logistical barriers and enabling simplified collection, transport, and storage [9].

Commercially DBS collection systems are available for general protein analysis, being Whatman filter paper cards the most commonly device used as alternative to serum samples in CD studies since the late 90s [10]. In addition to Whatman cards, other paper devices such as the Cobas Plasma Separation Cards (PSC) have been developed, which allow the collection and stabilization of dried plasma from whole blood. Currently, its use is widely recommended for HIV and HCV viral load monitoring [11,12].

The Elecsys Chagas assay, an automated electrochemiluminescence immunoassay (E-CLIA) for the determination of anti-*T. cruzi* antibodies, has demonstrated high sensitivity and specificity in serum [13] and in DBS samples collected on Whatman filter paper [14,15]. However, studies of PSC-collected samples have not been systematically evaluated for *T. cruzi* infection, but studies performed for hepatitis virus support the use of PSC device for serological assays [12,16].

The objective of this study was to assess the performance of blood sampling collected on Cobas Plasma Separation Cards and Whatman filter paper analyzed by the Elecsys Chagas assay for anti-*T. cruzi* antibody detection.

## Methods

### Ethical statement

Review Board approvals (PR(AG)409/2022) were obtained from the Ethics Committee of the Vall d’Hebron Research Institute, according to the principles expressed in the Declaration of Helsinki. All samples were anonymized before being analyzed; written informed consent for participation was not required for this study in accordance with the national legislation and the institutional requirements.

### Study samples and procedures

**Fig 1:**
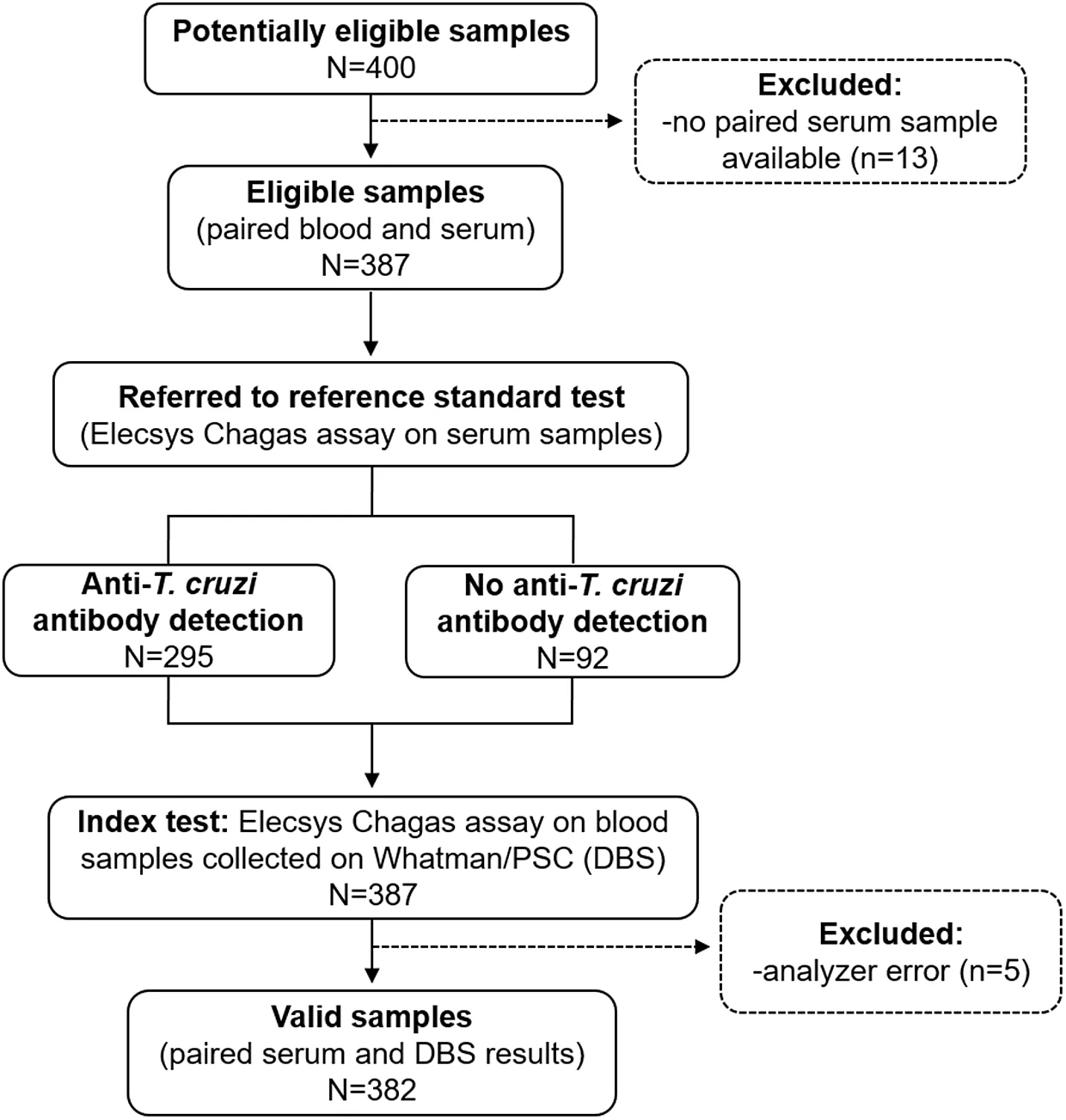
Study flowchart assessing Elecsys Chagas assay on Whatman/PSC-collected dried blood samples, according to the Standards for Reporting of Diagnostic Accuracy Studies (STARD) guidelines. PSC, cobas Plasma Separation Cards; DBS, dried blood spot.

A prospective agreement study was performed between July 2023 and May 2024 at the Microbiology Department of the Hospital Universitari Vall Hebron (HUVH) (Fig 1). Surplus paired serum and whole blood samples were used from adult patients attended at the International Health Unit of HUVH in whom serological testing for anti-*T. cruzi* antibodies was requested, regardless of whether it was an initial diagnosis or a follow-up visit. All serum samples were initially characterized for chronic *T. cruzi* infection according to WHO/PAHO recommendations using two serological assays based on different antigenic principles: Elecsys Chagas (recombinant antigen; Roche Diagnostics, Manheim, Germany) and Ortho *T. cruzi* ELISA (native antigen; Johnson & Johnson, High Wycombe, United Kingdom). However, for the purpose of matrix comparison, only Elecsys Chagas results were used, as it was the assay applicable to both serum and DBS samples.

Serum samples were stored at 4 °C until its analysis or -20 °C/-80 °C if the analysis was scheduled for more than one week after sample collection.

Whole blood samples were used to obtain DBS using two devices no more than 48 hours after blood collection: Whatman 903 Protein Saver Snap Apart Card (GE HealthCare, Chicago, IL) and Cobas Plasma Separation Cards (Roche Diagnostics).

Regarding to Whatman-collected samples, 100 µL of whole blood was spotted on two 8-mm-diameter circles (50 µL in each circle). Each card was dried overnight at room temperature. After that, two paper circles were placed in 600 μL of 1x phosphate-buffer saline (PBS) and incubated overnight with gentle rotation at room temperature. The paper was then carefully removed and the eluted blood spots were centrifuged for 2 minutes at 10500 x g to clear away any remaining paper from the supernatant and stored at 4 °C until analysis.

Regarding PSC-collected samples, 140 μL of whole blood was spotted on one spot of the PSC and dried overnight at room temperature. Each dried spot was removed from the cards and incubated overnight at 37°C in 600 μL of 1x PBS. Then, the samples were centrifuged at 113 x g for 10 min. The entire eluate was transferred to a new tube, and stored at 4 °C until analysis.

Whatman and PSC-collected samples were tested for the detection of anti-*T. cruzi* antibodies using the Elecsys Chagas assay (Roche Diagnostics) on the automated Cobas 8000 analyzer (Roche Diagnostics). Results for both serum and DBS samples were interpreted according to the Elecsys Chagas assay manufacturer’s cut-off values: samples with a cut-off index (COI) value of ≥1.0 were considered reactive (i.e., positive for antibodies to *T. cruzi*), and those with a COI value of <1.0 were considered non-reactive (i.e., negative for antibodies to *T. cruzi*).

### Statistical analysis

Sensitivity and specificity values were calculated to determine the diagnostic accuracy of the Elecsys Chagas on Whatman/PSC-collected samples against the reference test (Elecsys Chagas on serum samples). The Cohen Kappa coefficient was used to analyze the level of agreement between tests and interpreted as follows: slight (0.00-0.20), fair (0.21-0.40), moderate (0.41-0.60), substantial (0.61-0.80), and almost perfect agreement (0.81-1.00) [17].

A Bland-Altman plot was generated to assess the limits of agreement and the mean bias in the COI values obtained from the Whatman and PSC-collected samples.

Elecsys Chagas results from DBS samples were evaluated by a receiver operating characteristic (ROC) curve analysis. The optimum cut-off values for each DBS collection device on Elecsys Chagas assay were determined by maximizing Youden’s index [18].

Qualitative variables were expressed as absolute frequencies and percentages and quantitative variables as the median and interquartile range (IQR).

McNemar’s test was used for paired samples to analyze the differences between the qualitative results. Continuous variables were compared using the Mann-Whitney U test. Correlations between the COI values obtained from the Whatman and the PSC-collected samples were assessed using the Spearman’s statistical test. Differences between COI values obtained for Whatman and PSC-collected samples were assessed using the Wilcoxon signed-rank test. Significance was determined at a p-value <0.05 for all case.

Statistical analyses were done using SPSS Statistics v22.0 (IBMP Corp., Armonk, NY).

## Results

A total of 387 of DBS samples have been collected on both Whatman and PSC devices. Afterwards, 5 DBS samples collected on both Whatman and PSC were withdrawn from the study due to operator errors. Finally, a total of 382 samples had valid paired serum and DBS results.

Overall, Table 1 summarizes the results obtained for each type of sample collection device compared with the reference results.

**Table 1:** Comparison of IgG anti-T. cruzi detection in Whatman and PSC-collected samples versus serum samples using the Elecsys Chagas assay.

Sample collection device	Sample result	Serum samples				
		Positive	Negative	Sensitivity % (95% CI)	Specificity % (95% CI)	Kappa value (95% CI)
Whatman	Positive	279	0	95.55 (92.32-97.51)	100 (94.9-99.9)	0.910 (0.862-0.958)
	Negative	13	90			
PSC	Positive	277	0	94.86 (91.49-96.99)	100 (94.9-99.9)	0.897 (0.846-0.948)
	Negative	15	90			

PSC: Plasma Separation Card; CI, confidence interval.

The agreement between both Whatman and PSC-collected samples and the reference results was classified as almost perfect (kappa values >0.81). Nonetheless, significant statistical differences were observed in the classification of samples as positive or negative when comparing both Whatman and PSC-collected samples to the results obtained with serum samples (p < 0.001) (Table 1).

**Fig 2:**
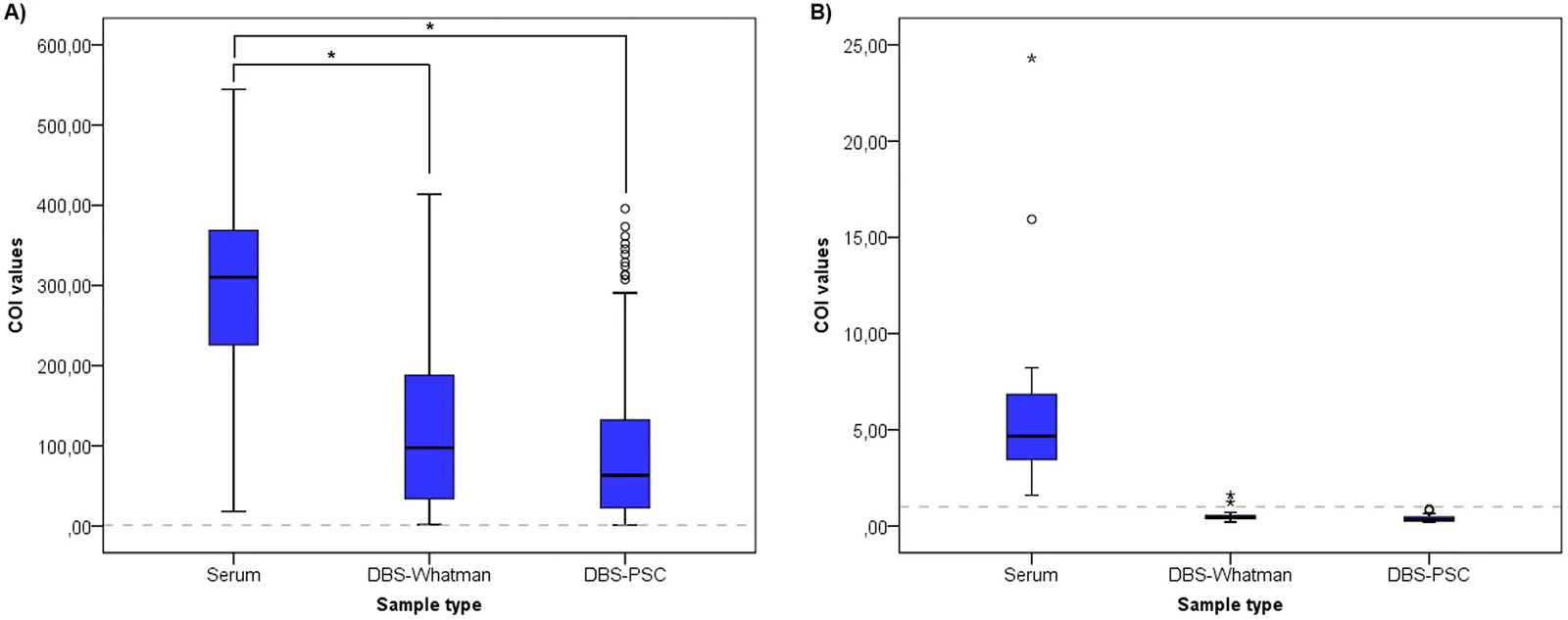
COI values obtained by the Elecsys Chagas assay according to the type of sample analyzed. **A)** Boxplot showing COI values in positive concordants samples (n = 277); **B)** Boxplot showing COI values in discordant samples (n = 13). Dashed lines indicate the cut-off value for the Elecsys Chagas on serum samples (≥1). COI: cut-off index; DBS: dried blood spot; PSC: plasma separation card. *, p < 0.05.

On the other hand, serum COI values were higher than those obtained on DBS samples (collected on Whatman and PSC); the median value for serum was 306.6 (IQR, 204.8 to 364.9), while the median value for Whatman-collected samples were 96.5 (IQR, 33.9 to 187.1; p < 0.05) and for PSC-collected samples was 63.3 (IQR, 22.5 to 132.2; p < 0.05) (Fig 2).

Compared with serum, 13/292 (4.45%) Whatman-collected samples and 15/292 (5.14%) PSC-collected samples were inappropriately classified as negative. COI values in the respective serum samples indicate that these discordant samples were associated with low IgG anti-*T. cruzi* levels [median serum COI, 4.67 (IQR, 3.45 to 6.84) vs. Whatman median, 0.41 (IQR, 0.36 to 0.49) and PSC median, 0.34 (IQR, 0.26 to 0.47)] (Fig 2).

**Fig 3:**
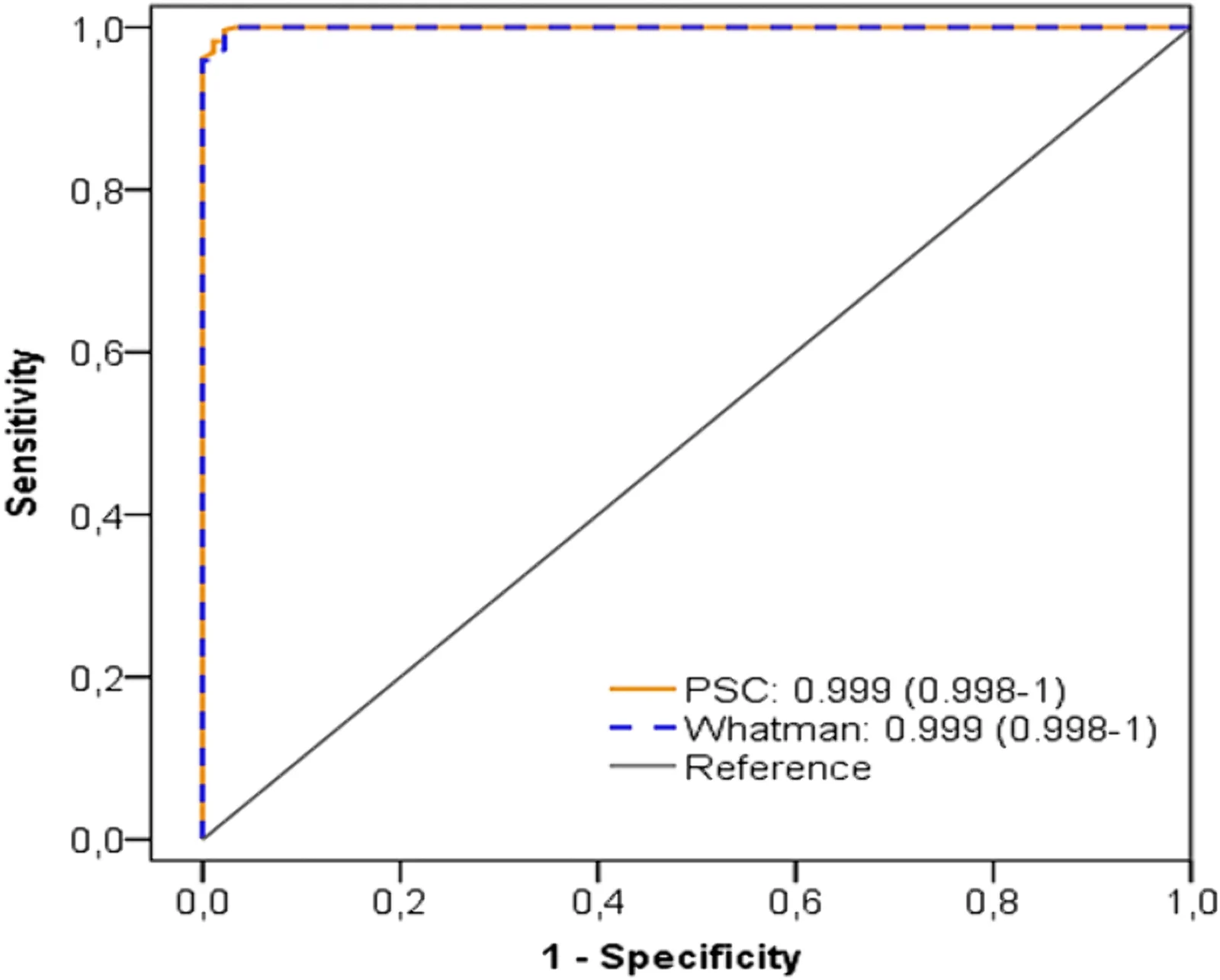
Multiple ROC constructed from the COI values obtained from Whatman and PSC-collected samples. The value of the area under the curve (95% confidence interval) is indicated for each device. PSC: plasma separation card.

DBS samples yielded low threshold (≥0.23 for PSC and ≥0.21 for Whatman) compared to the established serum cut-off (≥1) (Fig 3). When the optimal cut-off COI value was applied, the sensitivity increased to 100% and 99.7% for the Whatman and PSC collection devices, respectively, while the specifity decreased to 97.8% in both cases.

**Fig 4:**
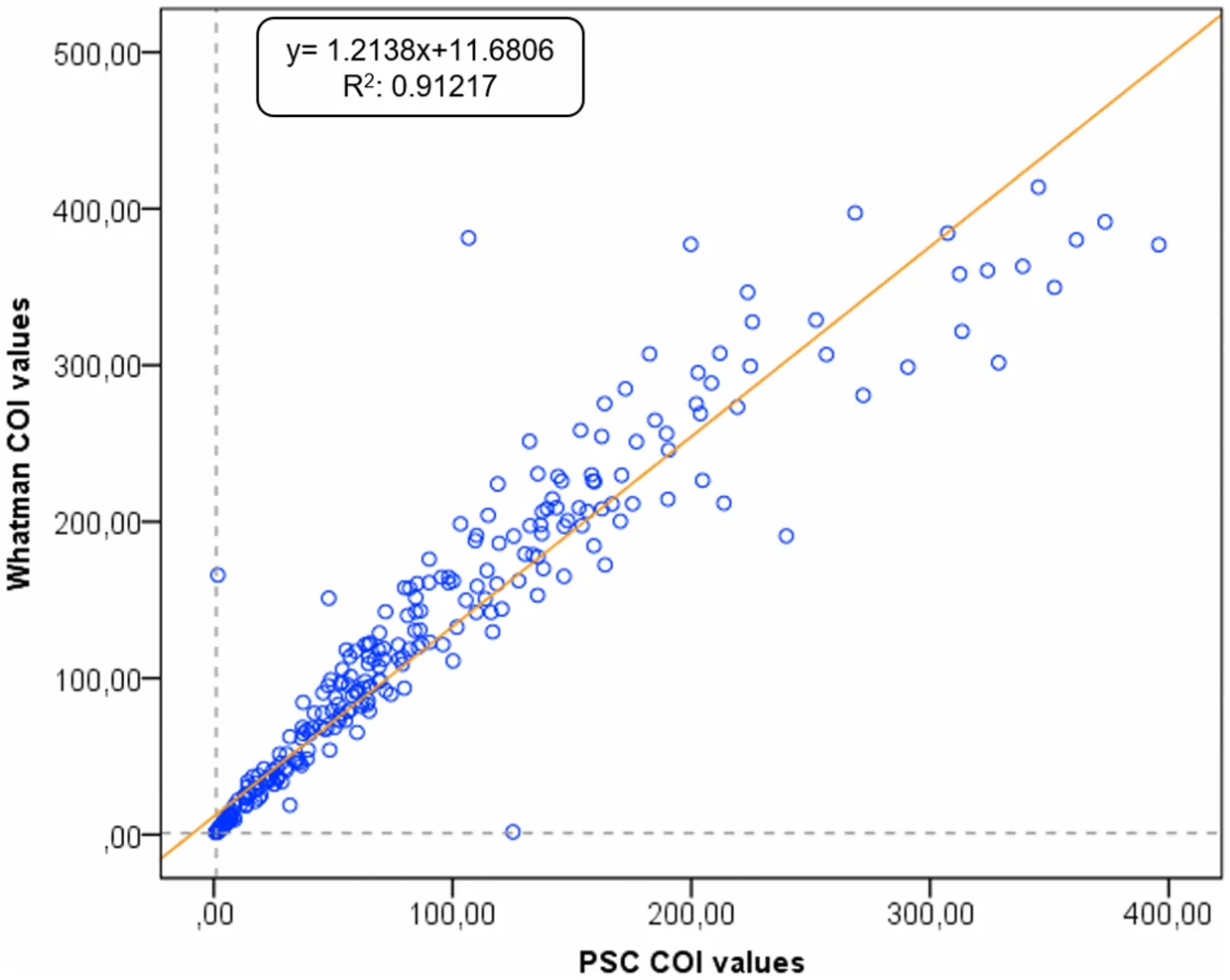
Distribution of COI values obtained by the Elecsys Chagas assay in each Whatman and PSC matched samples (n = 382). Linear regression equation and coefficient of determination (R^2^) are also displayed. Dashed lines indicate the cut-off value for the Elecsys Chagas on serum samples (≥1).

On the other hand, the classification of samples as positive or negative was highly consistent between PSC and Whatman devices. Out of 382 samples, 277 (72.5%) were classified as positive and 103 (27%) as negative by both methods, with only two (0.5%) discordant samples (both positive by Whatman and negative by PSC), resulting in almost perfect agreement (Kappa value: 0.987; p > 0.05). However, for each matched samples, COI values obtained from Whatman-collected samples were higher than those obtained with PSC (p < 0.001) (Fig 4). Despite this, a strong correlation between the two DBS sampling devices was observed, with a correlation coefficient of 0.974 (p < 0.0001) (Fig 4).

**Fig 5:**
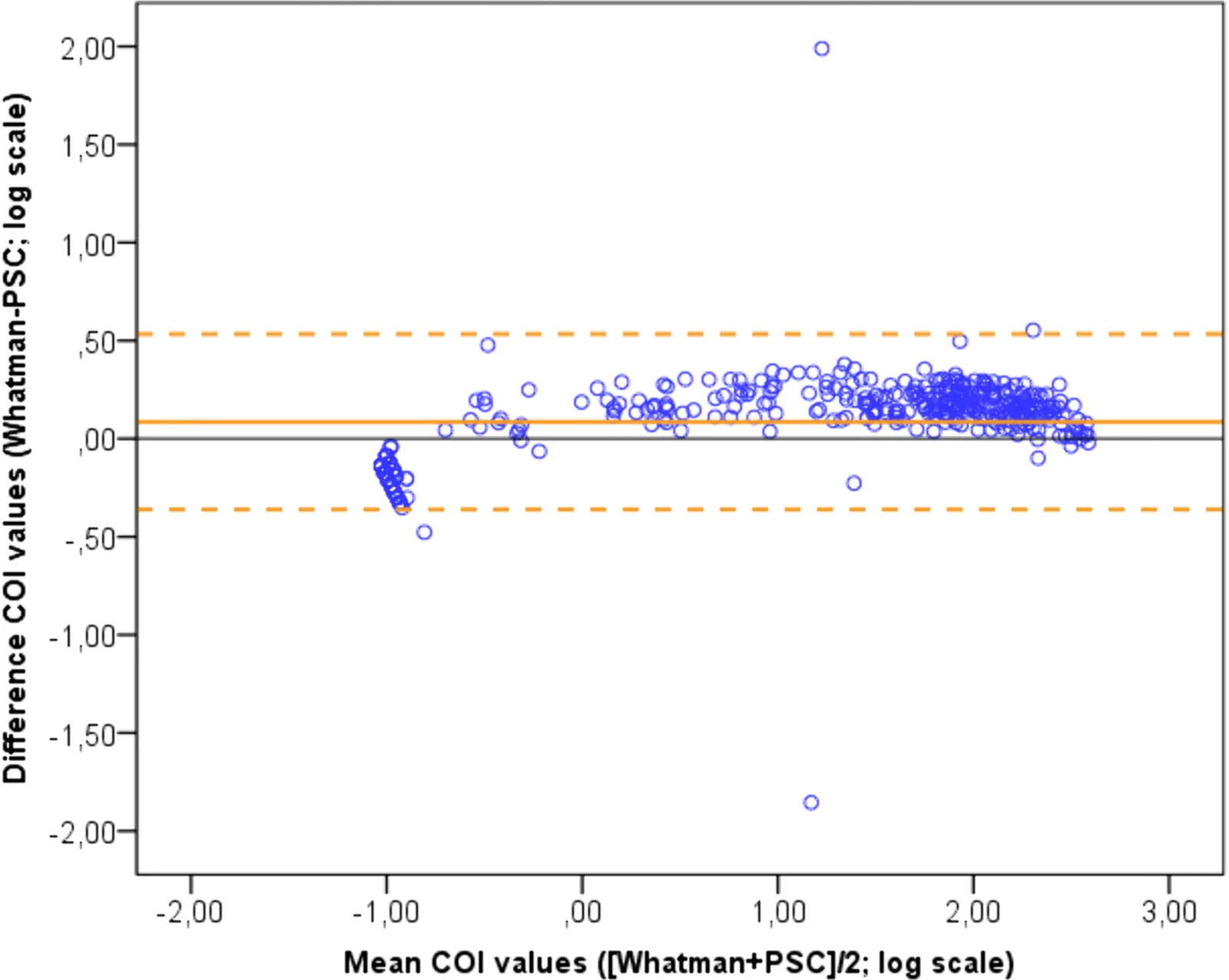
Bland-Altman analysis between Whatman and PSC sampling devices in anti-T. cruzi antibody detection using the Elecsys Chagas assay. Assay results are in log_10_ COI values. Orange solid line represents de mean difference and orange dashed lines show 95% limits of agreement.

The Bland-Altman analysis showed a bias of 0.09 and 95% limits of agreement of 0.53 to -0.36 log_10_ COI values; the total number of cases within the agreement limits was 379/382 (99.2%) (Fig 5).

## Discussion

Dried blood sampling on filter papers have been used in several studies for detecting antibodies against *T. cruzi*, particularly in endemic resource-limited or community surveys-based settings [19–24]. Most of the aforementioned studies utilized Whatman filter paper to evaluate DBS. To our knowledge, this study is the first to evaluate the use of PSCs for *T. cruzi* serological testing. Moreover, this studies have primarily used conventional serological tests for DBS analysis, such as inmunofluorescence or ELISA tests. However, validation of DBS samples with high-performance serological assays, such as E-CLIA, remains limited. Previously, studies conducted by Holguin et al [14] and Silgado et al [15] evaluated DBS collected on Whatman filter papers using CLIA assays, reporting high values of sensitivity (95–97%) and specificity (84–100%). Similar results have been obtained in the present study for both Whatman and PSC-collected samples, although the latter showed slightly lower sensitivity. As this is the first study reporting PSC sampling usefulness for *T. cruzi* serological testing in the published literature, comparisons with other studies are not possible.

Although the agreement between both DBS-collection devices and serum samples was classified as almost perfect (kappa values >0.81), significant differences were observed in the qualitative classification of samples. This underscores the importance of carefully validating DBS-based assays prior to their implementation in clinical or screening programs. Moreover, serum samples consistently yielded higher COI median values compared to DBS samples. This reduction in signal detection in DBS samples has been previously reported, generally attributed to factors such as sample volume collected and potential analyte loss during drying and elution protocols [25]. Lower COI values detected in DBS samples contributed to the misclassification of a small subset of samples as negative: 4.5% and 5.1% of Whatman and PSC-collected samples, respectively. These discordant samples exhibited low COI values in the respective serum, suggesting that the sensitivity of DBS-based assays may be compromised, specially in individuals with low antibody index values.

The optimization of cut-off values improved sensitivity (reaching 100% for Whatman and 99.7% for PSC) with minimal loss in specificity (97.8% for both devices). At their optimal cutt-off values, both sampling methods demonstrate excellent performance: with PSC-collected samples, only 1 positive case (false negative) was observed. However, both devices incorrectly classifies 2 negative cases (false positives). In the context of CD, where the primary concern in screening programs is to minimize false negatives, a higher sensitivity technique is generally preferred, even at the expense of a small reduction specifictiy, as all positive-cases should be subsequently confirmed [3,26]. In practice, these optimized DBS cut-offs values could serve as highly sensitive initial screening tools, followed by confirmatory serum testing to mitigate false positives. Nonthelesss, the Whatman device appears to have a slight advantage over the PSC device due to its perfect sensitivity while maintaining the same specificity.

On the other hand, the classification of samples as positive or negative for anti-*T. cruzi* antibodies was highly consistent between Whatman and PSC-collected samples, suggesting that both devices can be used interchangeably for chronic *T. cruzi* serological testing using Elecsys Chagas assay. However, COI values obtained from Whatman-collected samples were consistently higher than those from PSC. This reduced quantitative signal may affect antibody detection sensitivity, particularly in samples with low antibody index values, emphasizing the need for method optimization in different scenarios. While these findings suggest that the Whatman-collected samples might be preferable for use with Elecsys Chagas assay, operational factors should also be considered. In this regard, previous studies have shown that PSCs offer greater analyte stability under conditions of high temperature and humidity compared to Whatman paper, which may be advantageous for sample transport and storage in field settings where cold chain is not assured [27,28]. Furthermore, cost considerations are highly relevant for field implementation. In a cost-analysis study performed in a resource-limited setting, Nichols et al [29] reported lower costs per sample for Whatman-based DBS collection devices compared to PSC-collection. Overall, DBS sampling offers significant advantages over conventional serum collection due to its simple and minimally invasive collection process, simplified transport to laboratories equipped with Elecsys Chagas assay analyzers, and lower overall costs compared to serum tubes and the need for specialized personnel for venipuncture procedure [27]. Future studies including a formal cost-effectiveness analysis are still needed to determine the optimal approach for chronic *T. cruzi* screening programs in endemic regions.

As limitation, the present study was conducted under controlled laboratory conditions, which minimized analytical variability and ensures optimal handling of DBS samples. Notably, this is markedly different from field settings, where factors such as improper sample collection can compromise the viability of the technique [30]. Moreover, this approach was intentionally selected to assess the impact of DBS collection device on anti*-T. cruzi* antibody detection using the Elecsys Chagas assay. Therefore, the results should be interpreted in terms of agreement between DBS collection devices rather than as a measure of clinical diagnostic accuracy.

In summary, our analytical study demonstrates that DBS sampling, using both Whatman and PSC-collecting devices coupled with the Elecsys Chagas assay and optimized cut-off values, is a reliable and viable alternative to invasive serum sampling for the detection of anti-*T. cruzi* antibodies. This approach achieves high sensitivity and specificity, offers logistical and cost advantages, and could be particularly suitable for CD screening programs in settings where access to health facilities is limited. Notably, antibody index values were higher in Whatman-collected samples compared to PSC, which should be considered when selecting the collection device, especially for samples near the positivity cut-off. Further studies in endemic regions are recommended to confirm the adequacy of this method for large-scale screening programs.

## References

[1] World Health Organization. Chagas disease (American trypanosomiasis). 2026 [cited 25 Mar 2026]. Available: https://www.who.int/news-room/fact-sheets/detail/chagas-disease-(american-trypanosomiasis

[2] Pecoul B, Batista C, Stobbaerts E, Ribeiro I, Vilasanjuan R, Gascon J. The BENEFIT Trial: Where Do We Go from Here?. PLoS Negl Trop Dis. 2016. doi: 10.1371/journal.pntd.0004343 PMC4767872 26913759

[3] Pan American Health Organization (PAHO). Guidelines for the diagnosis and treatment of Chagas Disease. 2019.

[4] Ascanio LC, Carroll S, Paniz-Mondolfi A, Ramírez JD. In vitro diagnostic methods of Chagas disease in the clinical laboratory: a scoping review. Front Microbiol. 2024. doi: 10.3389/fmicb.2024.1393992 PMC11091413 38746745

[5] Pérez-Ayala A, Fradejas I, Rebollo L, Lora-Pablos D, Lizasoain M, Herrero-Martínez JM. Usefulness of the ARCHITECT Chagas® assay as a single test for the diagnosis of chronic Chagas disease. Trop Med Int Health. 2018;23(6):634–40. doi: 10.1111/tmi.13063 29683542

[6] Abras A, Ballart C, Fernández-Arévalo A, Llovet T, Gállego M, Muñoz C. ARCHITECT Chagas® as a single test candidate for Chagas disease diagnosis: evaluation of two algorithms implemented in a non-endemic setting (Barcelona, Spain). Clin Microbiol Infect. 2020. doi: 10.1016/j.cmi.2020.07.002 32653657

[7] Egüez KE, Alonso-Padilla J, Terán C, Chipana Z, García W, Torrico F, et al. Rapid diagnostic tests duo as alternative to conventional serological assays for conclusive Chagas disease diagnosis. PLoS Negl Trop Dis. 2017;11(4):e0005501. doi: 10.1371/journal.pntd.0005501 28369081 PMC5391121

[8] Perez F, Vermeij D, Salvatella R, Castellanos LG, de Sousa AS. The use of rapid diagnostic tests for chronic Chagas disease: An expert meeting report. PLoS Negl Trop Dis. 2024;18(8):e0012340. doi: 10.1371/journal.pntd.0012340 39116064 PMC11309409

[9] Parker SP, Cubitt WD. The use of the dried blood spot sample in epidemiological studies. J Clin Pathol. 1999;52(9):633–9. doi: 10.1136/jcp.52.9.633 10655983 PMC501537

[10] Machado-Coelho GL, Vitor RW, Chiari C de A, Antunes CM. Validity of serology for American trypanosomiasis with eluates from filter paper. Mem Inst Oswaldo Cruz. 1995;90(1):59–64. doi: 10.1590/s0074-02761995000100013 8524086

[11] World Health Organization. Consolidated guidelines on HIV prevention, testing, treatment, service delivery and monitoring: recommendations for a public health approach. Geneva: World Health Organization; 2021. 34370423

[12] Colucci G, Uceda Renteria S, Ceriotti F, Lampertico P. Clinical Evaluation of Plasma Separation Cards as a Tool to Collect, Store, and Test Blood Samples for Hepatitis B and C Serological Markers. Clin Chem. 2021;68(1):214–7. doi: 10.1093/clinchem/hvab170 34969104

[13] Flores-Chavez MD, Sambri V, Schottstedt V, Higuera-Escalante FA, Roessler D, Chaves M, et al. Evaluation of the Elecsys Chagas Assay for Detection of Trypanosoma cruzi-Specific Antibodies in a Multicenter Study in Europe and Latin America. J Clin Microbiol. 2018;56(5):e01446-17. doi: 10.1128/JCM.01446-17 29444836 PMC5925710

[14] Holguín A, Norman F, Martín L, Mateos ML, Chacón J, López-Vélez R, et al. Dried blood as an alternative to plasma or serum for Trypanosoma cruzi IgG detection in screening programs. Clin Vaccine Immunol. 2013;20(8):1197–202. doi: 10.1128/CVI.00221-13 23740927 PMC3754500

[15] Silgado A, Gual-Gonzalez L, Sánchez-Montalvá A, Oliveira-Souto I, Goterris L, Serre-Delcor N, et al. Analytical Evaluation of Dried Blood Spot and Rapid Diagnostic Test as a New Strategy for Serological Community Screening for Chronic Chagas Disease. Front Cell Infect Microbiol. 2021;11:736630. doi: 10.3389/fcimb.2021.736630 34604116 PMC8479190

[16] Velásquez-Orozco F, Rando-Segura A, Martínez-Camprecios J, Salmeron P, Najarro-Centeno A, Esteban À, et al. Utility of the Cobas® Plasma Separation Card as a Sample Collection Device for Serological and Virological Diagnosis of Hepatitis C Virus Infection. Diagnostics (Basel). 2021;11(3):473. doi: 10.3390/diagnostics11030473 33800211 PMC7998864

[17] Landis JR, Koch GG. The measurement of observer agreement for categorical data. Biometrics. 1977;33(1):159–74. doi: 10.2307/2529310 843571

[18] Ruopp MD, Perkins NJ, Whitcomb BW, Schisterman EF. Youden Index and optimal cut-point estimated from observations affected by a lower limit of detection. Biom J. 2008;50(3):419–30. doi: 10.1002/bimj.200710415 18435502 PMC2515362

[19] Montoya R, Dias JCP, Coura JR. Chagas disease in a community in southeast Brazil. I. A serologic follow-up study on a vector controlled area. Rev Inst Med Trop Sao Paulo. 2003;45(5):269–74. doi: 10.1590/s0036-46652003000500006 14743667

[20] Pinto FS, de Andrade GMQ, Januario JN, Maia MCA, Gontijo ED. Epidemiological profile of Trypanosoma cruzi-infected mothers and live birth conditions in the state of Minas Gerais, Brazil. Rev Soc Bras Med Trop. 2013;46(2):196–9. doi: 10.1590/0037-8682-1687-2013 23740060

[21] de Aquino Santana M, da Silva Ferreira AL, Dos Santos LVB, Furtado Campos JH, de Sena LLJ, Mendonça VJ. Seroprevalence of Chagas disease in rural communities at Campinas do Piauí city, Brazil. Trop Med Int Health. 2021;26(3):281–9. doi: 10.1111/tmi.13516 33159709

[22] Moscatelli G, Berenstein A, Tarlovsky A, Siniawski S, Biancardi M, Ballering G, et al. Urban Chagas disease in children and women in primary care centres in Buenos Aires, Argentina. Mem Inst Oswaldo Cruz. 2015;110(5):644–8. doi: 10.1590/0074-02760150107 26222020 PMC4569828

[23] Russomando G, Cousiño B, Sanchez Z, Franco LX, Nara EM, Chena L, et al. Chagas disease: national survey of seroprevalence in children under five years of age conducted in 2008. Mem Inst Oswaldo Cruz. 2017;112(5):348–53. doi: 10.1590/0074-02760160407 28443980 PMC5398161

[24] Pérez-Sánchez E, Montiel-Cruz R, Romero-Domínguez E, Pascacio-Bermúdez G, Báez-Hernández A, Díaz Del Castillo-Flores G, et al. Seroprevalence of Trypanosoma cruzi among children from Veracruz, Mexico: Epidemiological baseline for a control model based on Chagas disease active transmission. Biomedica. 2024;44(1):92–101. doi: 10.7705/biomedica.7126 38648342 PMC11204380

[25] McClendon-Weary B, Putnick DL, Robinson S, Yeung E. Little to Give, Much to Gain-What Can You Do With a Dried Blood Spot? Curr Environ Health Rep. 2020;7(3):211–21. doi: 10.1007/s40572-020-00289-y 32851603 PMC7500853

[26] Sulleiro E, Flores M, Lozano N, Navarro M, Sulleiro E, Trigo E. Enfermedad de Chagas. Documentos cortos Grupo de Estudio de Patología Importada (GEPI). 2021.

[27] Amini F, Auma E, Hsia Y, Bilton S, Hall T, Ramkhelawon L. Reliability of dried blood spot (DBS) cards in antibody measurement: A systematic review. PLoS ONE. 2021. doi: 10.1371/journal.pone.0248218 PMC7959368 33720928

[28] Roche Molecular Systems. cobas Plasma Separation Card. Basel; 2017 [cited 2 Jul 2025]. Available: https://molecular.roche.com/about/global-access-program/

[29] Nichols BE, Girdwood SJ, Shibemba A, Sikota S, Gill CJ, Mwananyanda L, et al. Cost and Impact of Dried Blood Spot Versus Plasma Separation Card for Scale-up of Viral Load Testing in Resource-limited Settings. Clin Infect Dis. 2020;70(6):1014–20. doi: 10.1093/cid/ciz338 31321438 PMC7931834

[30] Silgado A, Bosch-Nicolau P, Sánchez-Montalvá A, Cervià A, Gomez-I-Prat J, Bagaria G, et al. Opportunistic Community Screening of Chronic Chagas Disease Using a Rapid Diagnosis Test in Pharmacies in Barcelona (Catalonia, Spain): Study Protocol and Pilot Phase Results. Int J Public Health. 2022;67:1605386. doi: 10.3389/ijph.2022.1605386 36531607 PMC9747760

